# The sounds go boom but then what?

**DOI:** 10.1093/conphys/coz057

**Published:** 2019-09-26

**Authors:** Kim Birnie-Gauvin

**Affiliations:** Technical University of Denmark, Vejlsøvej 39, Silkeborg, Denmark

Did you know that, in order to obtain oil and gas from the ocean floor, industries first explore the territory with seismic airguns? These airguns create some of the loudest human-made sounds in the oceans—and can you guess what else happens? They do not just fire the airguns once; the average seismic survey consists of 7000 shots over 24 hours! How does the marine life cope with such noise?

The sounds of airguns are equivalent to 140–180 dB on land. To put things into perspective, 140 dB is the pain threshold in humans, 150 decibels is like standing right next to the speakers at a Rammstein concert and 180 dB is like being 3 m away from a couple kilos of dynamite exploding. You know the famous movie scenes where the action hero is seemingly paralysed after witnessing an explosion? Their heart is racing and they cannot hear anything? Perhaps fish are similarly affected. In a new study, Davidsen et al. (2019) investigated just that. They wondered how cod and saithe—two culturally and economically important species—respond to the sound of seismic airguns.

Davidsen’s team used biologgers to record heart rate and body temperature and acoustic tags to record activity and depth of the fish. Biologgers are miniaturized tags attached to or surgically implanted inside of animals that can log information about the animal’s physiology, behaviour and/or environment. Acoustic tags are attached to the animal and emit sounds at specific intervals that can be detected by hydrophones. These sound detections can then be used to determine an animal’s position when combined with depth recordings. With all of this gear, the team took to a fjord in Norway and performed a controlled experiment on fish that were free-swimming but confined in a large sea cage. After some baseline measurements, the team exposed the fish to sounds ranging 130–180 dB over a 3-day period.

The sounds resulted in a short-lived decrease in heart rate in cod, which the authors suggest reflected the initiation of the fight-or-flight response. While neither fish species startled upon exposure to the noise, both species changed their swimming depth and horizontal position more often during the sound exposure than when it was quiet. Saithe also dispersed from one another during the noise exposure, which is not a typical behaviour for saithe—some of the strongest schooling fish known. Over time though, it seems that both fish species habituated to repeated noise exposure, both in their physiology and behaviour.

So what does this tell us? Well at this point, it remains unknown just how bad these seismic surveys are for the marine ecosystem as a whole, but it seems that, at least for cod and saithe, exposure to airgun sounds for a 3-day period is unlikely to have long-term consequences. Maybe, despite the noise, the fish still felt safe, given that they were maintained in large sea cages during the experiment. In the wild, the observed changes in swimming depth and schooling behaviour could have profound consequences on both energy and habitat use. More investigation is surely needed.

Illustration by Erin Walsh; Email: ewalsh.sci@gmail.com


